# Conductive nanocomposite hydrogel and mesenchymal stem cells for the treatment of myocardial infarction and non-invasive monitoring via PET/CT

**DOI:** 10.1186/s12951-022-01432-7

**Published:** 2022-05-06

**Authors:** Ke Zhu, Dawei Jiang, Kun Wang, Danzha Zheng, Ziyang Zhu, Fuqiang Shao, Ruijie Qian, Xiaoli Lan, Chunxia Qin

**Affiliations:** 1 Department of Nuclear Medicine, Union Hospital, Tongji Medical College, Huazhong University of Science and Technology, No. 1277 Jiefang Ave, Wuhan, 430022 Hubei China; 2 Hubei Key Laboratory of Molecular Imaging, Wuhan, 430022 China

**Keywords:** Myocardial infarction, Gold nanorods, Mesenchymal stem cells, Conductive hydrogel, Positron emission tomography (PET)

## Abstract

**Background:**

Injectable hydrogels have great promise in the treatment of myocardial infarction (MI); however, the lack of electromechanical coupling of the hydrogel to the host myocardial tissue and the inability to monitor the implantation may compromise a successful treatment. The introduction of conductive biomaterials and mesenchymal stem cells (MSCs) may solve the problem of electromechanical coupling and they have been used to treat MI. In this study, we developed an injectable conductive nanocomposite hydrogel (GNR@SN/Gel) fabricated by gold nanorods (GNRs), synthetic silicate nanoplatelets (SNs), and poly(lactide-*co*-glycolide)-*b*-poly (ethylene glycol)-*b*-poly(lactide-*co*-glycolide) (PLGA-PEG-PLGA). The hydrogel was used to encapsulate MSCs and ^68^Ga^3+^ cations, and was then injected into the myocardium of MI rats to monitor the initial hydrogel placement and to study the therapeutic effect via ^18^F-FDG myocardial PET imaging.

**Results:**

Our data showed that SNs can act as a sterically stabilized protective shield for GNRs, and that mixing SNs with GNRs yields uniformly dispersed and stabilized GNR dispersions (GNR@SN) that meet the requirements of conductive nanofillers. We successfully constructed a thermosensitive conductive nanocomposite hydrogel by crosslinking GNR@SN with PLGA_2000_-PEG_3400_-PLGA_2000_, where SNs support the proliferation of MSCs. The cation-exchange capability of SNs was used to adsorb ^68^Ga^3+^ to locate the implanted hydrogel in myocardium via PET/CT. The combination of MSCs and the conductive hydrogel had a protective effect on both myocardial viability and cardiac function in MI rats compared with controls, as revealed by ^18^F-FDG myocardial PET imaging in early and late stages and ultrasound; this was further validated by histopathological investigations.

**Conclusions:**

The combination of MSCs and the GNR@SN/Gel conductive nanocomposite hydrogel offers a promising strategy for MI treatment.

**Graphical Abstract:**

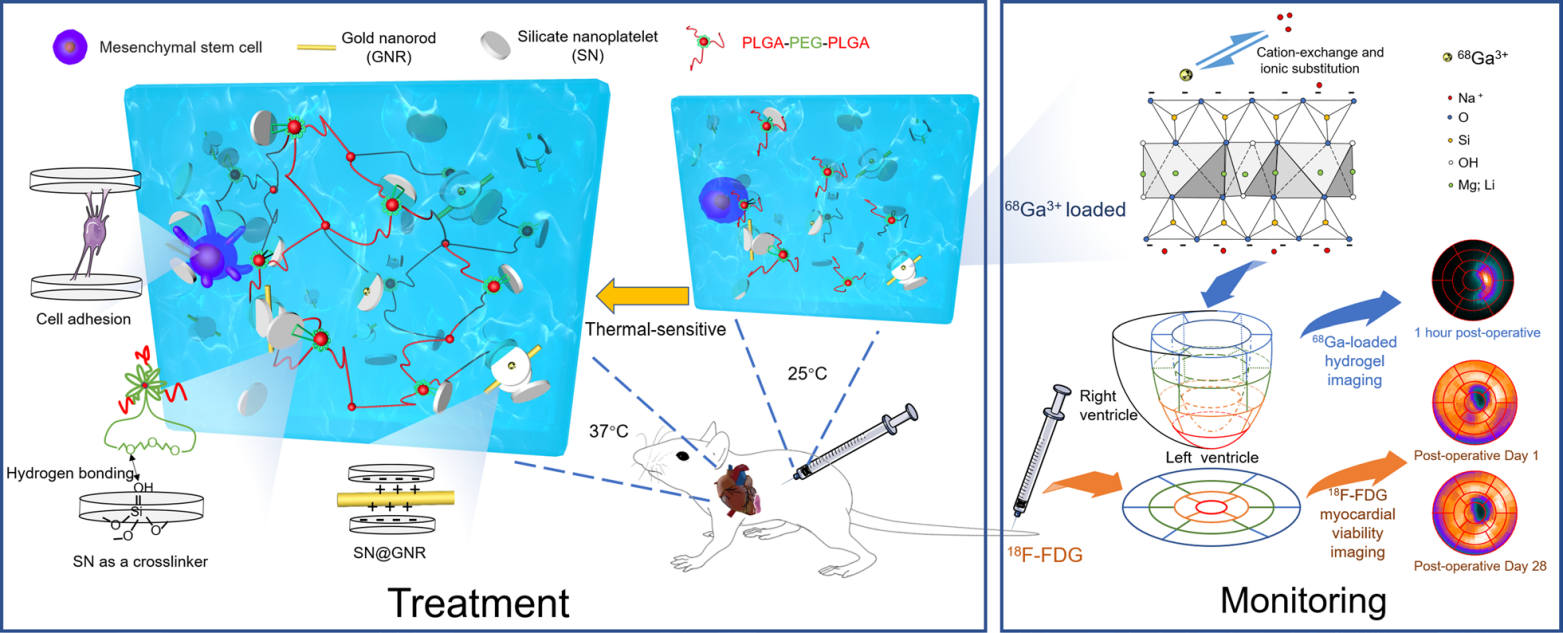

**Supplementary Information:**

The online version contains supplementary material available at 10.1186/s12951-022-01432-7.

## Background

Myocardial infarction (MI), induced by occluded coronary arteries, is the primary cause of cardiovascular mortality worldwide. MI results in ischemic death of the myocardium and progressive negative ventricular remodeling, the resulting high mortality calls for the timely intervention of MI [1].

Intramyocardial injectable hydrogels [2, 3] can provide mechanical support for damaged cardiac structures, improve the compliance of the myocardial wall, and serve as platforms for the controlled local delivery of therapeutic agents or cells to the damaged area of the heart. However, the absence of electromechanical coupling of the hydrogel to the host myocardial tissue is among challenges that can compromise the success of injectable hydrogel-based therapies [4].

Mesenchymal stem cells (MSCs) are stromal cells that can be isolated from a variety of tissues such as bone marrow or the umbilical cord. MSCs can repair injured tissues by self-renewal, differentiation, and other diverse mechanisms [5]. MSCs exhibit promising anti-fibrosis potential by inhibiting fibrosis development and progression, and optimizing fibrotic scar formation [6–9]. Furthermore, gap junctions are structures consisting of connexon protein that mediates electrical conduction in cardiomyocytes, an important feature of MSCs that can express connexon 43 (CX43) protein and form functional gap junctions with cardiomyocytes [10, 11]. Excessive deposition of collagen and loss of functional cardiomyocytes weaken linear electrical propagation and leads to arrhythmia because of the reduced level of CX43 [12]. Introducing MSCs into the infarction region may allow for regulation of arrhythmia substrates and improve electromechanical coupling with the host myocardium by achieving a syncytial structure [10, 13].

The electrical pulse signal in the infarct region is interdicted following the occurrence of MI, and the introduction of electrical conductivity into the biomaterials to facilitate electrical propagation across an infarct region is an effective approach to promote cardiac function after MI [14, 15]. In cardiac tissue engineering, MSCs can be guided to differentiate toward cardiomyocyte-like cells via conductive materials, specifically showing the up-regulation of CX43 [16]. Thus, combining conductive materials with MSCs may play a combinatorial role in the treatment of MI [16, 17]. In fact, the use of conductive hydrogel encapsulating stem cells for the treatment of MI has been reported with good efficacy [17]; however, there is a lack of understanding of the specific roles played by each component (MSCs and conductive materials) during the treatment process. Therefore, in this work, we designed a novel cell-loadable conductive hydrogel for the treatment of MI, and comprehensively monitored its treatment efficacy.

**Fig. 1:**
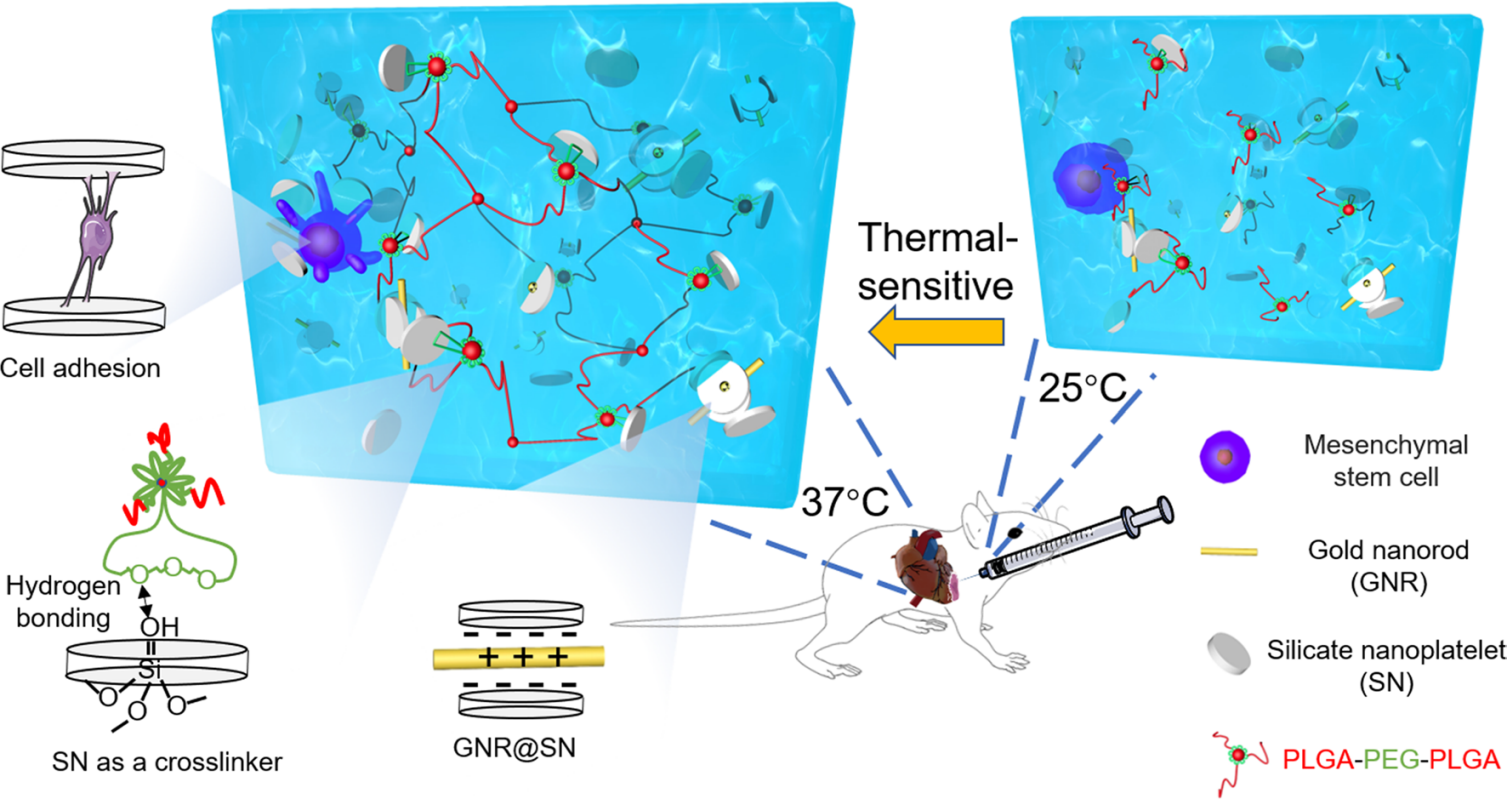
Schematic illustration of the MSC-loaded conductive hydrogel for cardiac repair. Injectable conductive nanocomposite hydrogel (GNR@SN/Gel) fabricated by GNRs, SNs, and PLGA-PEG-PLGA was combined with MSCs to treat myocardial infarction. GNRs act as a conductive component in hydrogel, SNs act as a crosslinker of hydrogel and a stabilizing agent of GNRs, and provide adhesion of hydrogel to MSCs. The thermal sensitive GNR@SN/Gel has a critical transition temperature of 27 °C, so it was flowable sol phase at 25 °C and transformed into a gel at 37 °C

For injectability and cell encapsulation, our preference is to design a smart hydrogel that encapsulates cells in the liquid phase and converts to the gel phase in situ after injection into the heart. PLGA-PEG-PLGA is a widely used thermal-sensitive smart hydrogel that is easy to synthesize, and different molecular weights of the tri-block molecules create different sol–gel phase transition temperatures [18]; however, it does not support cell adhesion and it disrupts cell membranes during the phase transition, leading to cell death [19, 20]. Silicate nanoplatelets (Laponite^®^ XLG, Mg_5.34_Li_0.66_Si_8_O_20_(OH)_4_Na_0.66_, hereafter referred to as SNs) as a crosslinker may reduce the critical micelle concentration of PLGA-PEG-PLGA, thus achieving reduced PLGA-PEG-PLGA hydrogel damage to the loaded cells [20–22]. In addition, the introduction of SNs into the hydrogel can achieve adhesion to cells by adsorbing organic macromolecules [21]. In this study, we fabricated a cell-loadable thermal-sensitive conductive hydrogel by PLGA-PEG-PLGA, SNs and gold nanorods (GNRs) (Fig. 1).

Epicardial injections and minimally invasive catheter approaches have been clinically explored for intramyocardial delivery of hydrogels [23, 24]. In either delivery approach, the efficacy of the hydrogel as an implant is related to the correct implantation site, and it is more difficult because of the continuous beating state of the heart during treatment. We took advantage of the principle that SN cation exchange can adsorb large-diameter and high-valent cations, and added ^68^Ga^3+^ to the hydrogel to monitor the injection sites of the hydrogel. An innovative method is proposed to locate the myocardium segments implanted by hydrogel.

This work has two goals: construction of a cell-loadable conductive smart hydrogel for MI treatment, and noninvasively monitoring treatment and efficacy evaluation using PET/CT in vivo.

## Results

### Characterization of the injectable nanocomposite hydrogel

**Fig. 2:**
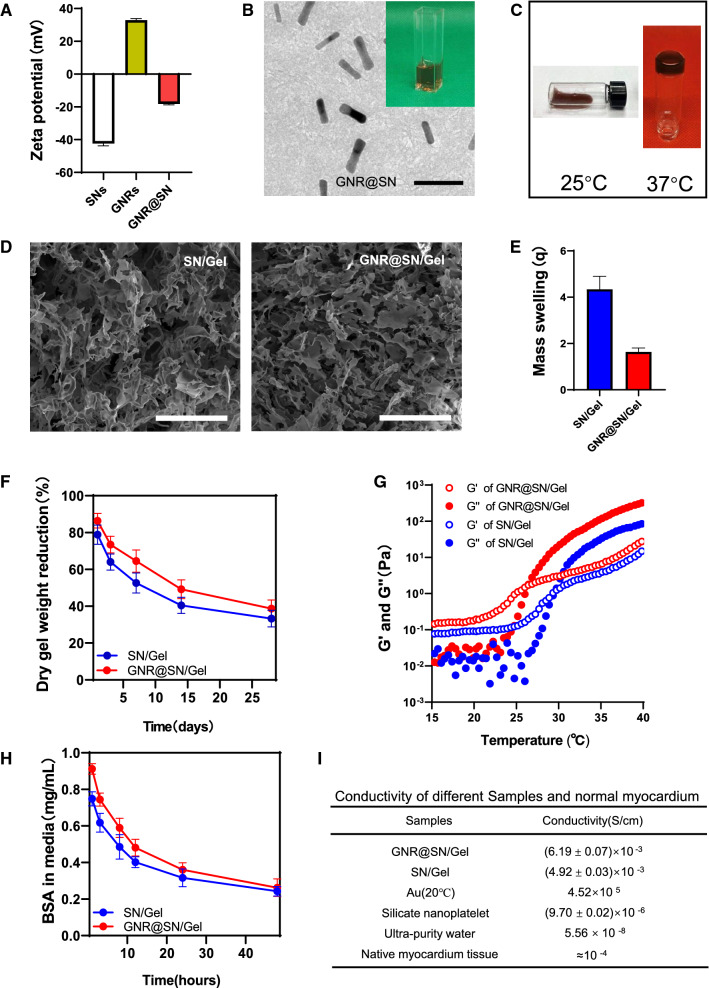
Characterization of the nanoparticles and nanocomposite injectable hydrogel. **A** Zeta potential of silicate nanoplatelets (SNs), gold nanorods (GNRs), and GNR@SN. **B** TEM image of GNR@SN (Scale bar: 50 nm). The insert is GNR@SN mixed with PBS after 1 day of resting. **C** The sol–gel transformation of GNR@SN/Gel. **D** SEM images of SN/Gel and GNR@SN/Gel (Scale bar: 50 μm). **E** Swelling ratio of SN/Gel and GNR@SN/Gel. **F** Dry gel weight reduction of SN/Gel and GNR@SN/Gel. **G** Temperature dependence of the storage modulus (G′) and loss modulus (G″) of SN/Gel and GNR@SN/Gel. **H** BSA adsorption test of SN/Gel and GNR@SN/Gel. **I** Conductivities of different samples

Figure 2A shows the zeta potentials of GNR, SN, and GNR@SN composite dispersions [1% (w/v) SN, 0.3% (w/v) GNR]. TEM image of the GNR@SN composite dispersions demonstrates GNRs stabilized in SN with good dispersity (Fig. 2B). Next, phosphate buffered saline (PBS) was added to the GNR@SN composite dispersion or naked GNR dispersion. GNR@SN composite dispersion didn’t have agglomerated particles (Fig. 2B), while the naked GNR dispersion had agglomerated particles [25, 26].

An inverted test tube experiment was used to measure the phase transition temperature of the hydrogel. A polymer of PLGA_2000_-PEG_3400_-PLGA_2000_ (characterization results are shown in Additional file 1: Fig. S1) didn’t form a sol–gel transition without a crosslinker; however, when the crosslinker SNs was added to formulate SN/Gel [3% (w/v) PLGA_2000_-PEG_3400_-PLGA_2000_, 1% (w/v) SNs], the critical transition temperature was 31 °C, the hydrogel precursor transformed into a gel at 37 °C, with a flowable sol phase at 28 °C (Additional file 1: Fig. S2). When the GNR@SN composite dispersion was added to create GNR@SN/Gel [3% (w/v) PLGA_2000_-PEG_3400_-PLGA_2000_, 1% (w/v) SN, 0.3% (w/v) GNR], the critical transition temperature was 27 °C, the hydrogel precursor transformed into a gel at 37 °C, with a flowable solid phase at 25 °C (Fig. 2C).

The SEM image shows that both SN/Gel and GNR@SN/Gel formed a 3D network-like structure, GNR@SN/Gel appears to be denser than SN/Gel (Fig. 2D). Changes in the hydrogel crosslink density and porosity can alter several other hydrogel properties, e.g., modulus and diffusivity [27, 28]. GNR@SN/Gel exhibited poor swelling properties (Fig. 2E), a slower degradation rate (Fig. 2F) and better mechanical properties than SN/Gel (Fig. 2G). The GNR@SN/Gel has a lower critical transition temperature than the SN/Gel (Additional file 1: Fig. S2), which is consistent with the rheological test results (Fig. 2G).

Because PEG, PLGA, and GNRs are biologically inert to bioactive protein adsorption/retention and cell proliferation, the presence of SNs can enhance cell adhesion and proliferation on the gel surface [21]. In experiments on the adsorption of organic substances from the hydrogel, GNR@SN/Gel adsorbed bovine albumin (BSA) more slowly than SN/Gel; however, there was no difference in the total amount of BSA adsorbed by GNR@SN/Gel and SN/Gel at 48 h (Fig. 2H). It is possible that the lower porosity of GNR@SN/Gel affected the diffusivity of BSA from the solution to the hydrogel; however, we did not observe an effect of GNR on the adsorption capacity of SN.

Finally, we tested the conductivity of samples (Fig. 2I). The conductivity of GNR@SN/Gel [(6.19 ± 0.07) × 10^−3^ S/cm] is significantly higher than that of SN/Gel [(4.92 ± 0.03) × 10^−3^ S/cm, *P* < 0.0001], both exceeding that of natural myocardial tissue. But the conductivity of SN powder was only 9.70 ± 0.04 × 10^–6^ when squeezed into blocks at 30 MPa.

### Viability of MSCs loaded inside the conductive hydrogel

**Fig. 3:**
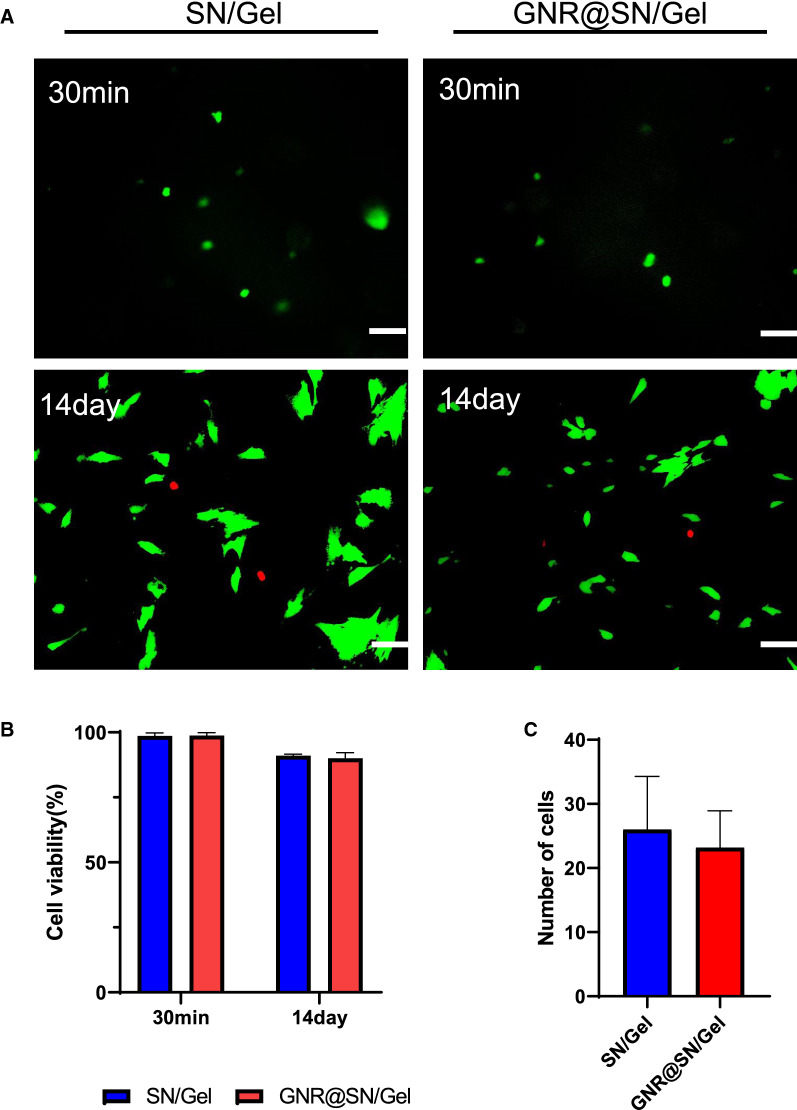
Viability of mesenchymal stem cells (MSCs) loaded inside the hydrogels. **A** Live/dead assay of the MSCs encapsulated in SN/Gel and GNR@SN/Gel at 30 min and 2 weeks. Living cells were stained with calcein AM (green) and dead cells were stained with PI (red) (Scale bar: 50 µm). **B** Quantitative analysis of the cell viability. **C** Microscopic calculations of encapsulated cells in hydrogels on Day 14

Flow cytometry analysis revealed that the extracted MSCs had high purity after three generations of culture (Additional file 1: Fig. S3). The biocompatibility of the hydrogel was determined by the viability assay of the encapsulated cells. MSCs were readily encapsulated in SN/Gel and GNR@SN/Gel via thermal gelation, 98.6% ± 1.2% and 98.8% ± 1.1% of the cells were alive within 30 min of gel formation (Fig. 3A), respectively, with no significant difference between two groups. Cells encapsulated in SN/Gel and GNR@SN/Gel exhibited continuous proliferation over 14 days, the percentage of live cells was 90.06% ± 4.74% for the GNR@SN group and 91.00% ± 0.39% for the SN/Gel group on day 14 (Fig. 3B). No significant difference was observed microscopically (Fig. 3C).

### Monitoring of injection location and myocardial viability by PET after treatment

**Fig. 4:**
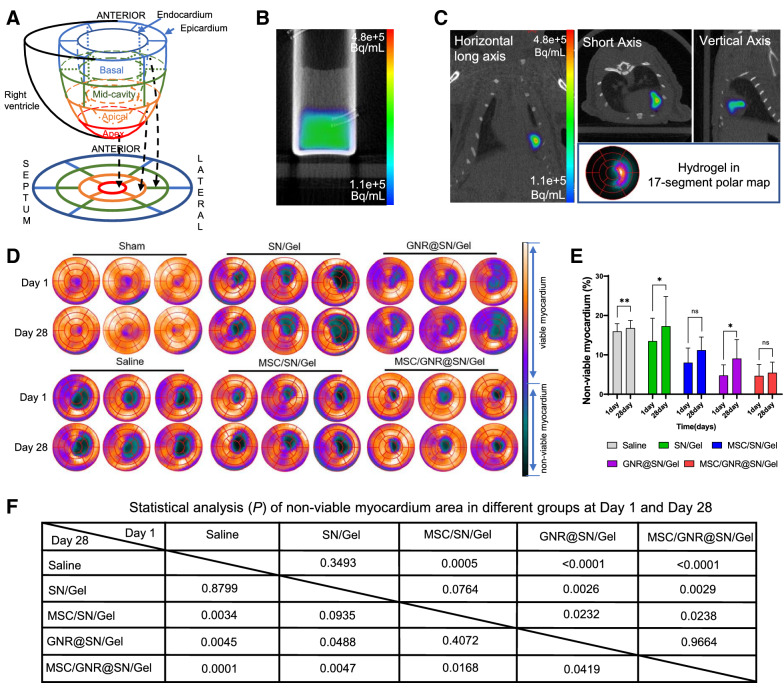
Non-invasive monitoring treatment and efficacy assessment through PET imaging. **A** Left ventricle is divided into 17 segments according to the AHA 17-segment model. **B** PET imaging of ^68^Ga-loaded GNR@SN/Gel hydrogel with PBS in vitro. **C** Region of hydrogel injection into myocardium visualized by ^68^GaCl_3_ PET. **D** Representative 17-segment polar map via ^18^F-FDG PET imaging of myocardial viability in each group after 1 day and 4 weeks. **E** Quantification of non-viable myocardium area as a percentage of total left ventricular area; **F** Comparison of non-viable myocardium between groups. *P < 0.05 and **P < 0.01, ***P < 0.001, ****P < 0.0001, ns > 0.05

Thirty-six rats (n = 6 per group) were divided into 6 random groups. Rats in the Sham group underwent sham surgery without MI operation. The remaining 5 groups underwent MI operation and received intramyocardial injection at the infarct region: saline alone (Saline group), PLGA-PEG-PLGA hydrogel precursor with SNs (SN/Gel group), MSCs-encapsulated PLGA-PEG-PLGA hydrogel precursor with SNs (MSC/SN/Gel group), GNRs stabilized on the SNs with PLGA-PEG-PLGA hydrogel precursor (GNR@SN/Gel group), and MSCs-encapsulated PLGA-PEG-PLGA hydrogel precursor with the GNRs stabilized on the SNs (MSC/GNR@SN/Gel group). Left ventricles were divided into 17 segments according to the AHA 17-segment model (Fig. 4A).

First, a release test of the ^68^Ga-loaded hydrogel was performed. As shown in Additional file 1: Fig. S4, GNR@SN/Gel hydrogel released 4.73% ± 1.65% ^68^Ga^3+^ after 1 h, in vitro PET imaging of ^68^Ga-loaded GNR@SN/Gel hydrogel shows very low activity in the PBS (Fig. 4B), demonstrating tight ^68^Ga^3+^ binding to the hydrogel, and it is possible to visualize the hydrogel injected into the heart in vivo by loading ^68^Ga^3+^ (Fig. 4C). The PET imaging of the hydrogel in rats was processed to obtain a 17-segment polar map. The region where the hydrogel injected was compatible with the region of non-viable myocardium induced by MI operation (Fig. 4C, D).

Next, ^18^F-FDG-PET imaging was performed to investigate the effect of MSCs combined with GNR@SN/Gel on the myocardial viability. As shown in Fig. 4D, ^18^F-FDG uptake in the myocardium of Sham group was uniform except low uptake at the apex segment, which was still greater than 40% of the highest value of the whole heart uptake, indicating no non-viable myocardium. On the first day after MI operation, the area percentages of non-viable myocardium were 15.95 ± 1.97, 13.45 ± 5.82, 8.12 ± 3.24, 4.75 ± 2.73, 4.68 ± 2.89 in the Saline group, SN/Gel group, MSC/SN/Gel group, GNR@SN/Gel group and MSC/GNR@SN/Gel group, respectively. The non-viable myocardium area of MSC/GNR@SN/Gel group and GNR@SN/Gel group was significantly lower than other groups (*P* < 0.05), however, no significant deference was found between GNR@SN/Gel group and MSC/GNR@SN/Gel group (*P* = 0.9664). These results indicate that GNR@SN/Gel and MSC/GNR@SN/Gel had a protective effect on the myocardial viability at an early stage.

On the 28 day after MI operation, the area percentages of non-viable myocardium were 16.79 ± 1.95, 17.28 ± 7.52, 11.14 ± 3.07, 9.13 ± 4.76, 5.47 ± 2.70 in the Saline group, SN/Gel group, MSC/SN/Gel group, GNR@SN/Gel group and MSC/GNR@SN/Gel group, respectively. GNR@SN/Gel group and SN/Gel group did not show a sustained protective effect on myocardial viability, whereas MSC/SN/Gel group and MSC/GNR@SN/Gel group showed an inhibitory effect on the spread of non-viable myocardium. The non-viable myocardium area of MSC/GNR@SN/Gel group was significantly lower than other groups.

Paired t-test was performed to compare the area of non-viable myocardium of each group on day 1 and day 28. The results showed Saline group, SN/Gel group and GNR@SN/Gel group were statistically different (*P* = 0.029,* P* = 0.013), while no statistical difference in MSC/GNR@SN/Gel group and MSC/SN/Gel group (*P* = 0.082, *P* = 0.353). The non-viable myocardium area in the MSC/SN/Gel group was significantly larger than that in MSC/GNR@SN/Gel group, indicating that the MSC/GNR@SN/Gel group had the best effect of inhibiting the enlargement of non-viable myocardium, and in the other groups, the inhibition of non-viable myocardium enlargement was weaker or did not last as long as MSC/GNR@SN/Gel group (Fig. 4E).

### Evaluation of heart function by echocardiography

**Fig. 5:**
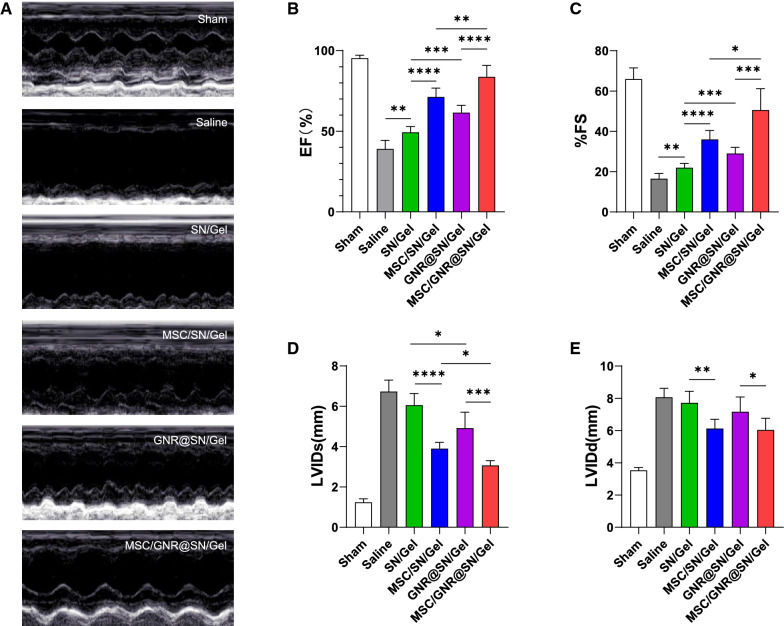
Cardiac function evaluated by echocardiography at postoperative Day 28. **A** Representative echocardiography images of different groups. **B** Left ventricle ejection fraction (EF). **C** Fractional shortening (FS). **D** The left ventricular internal diameter at end-systole (LVIDs). **E** The left ventricular internal diameter at end-diastole (LVIDd). *P < 0.05 and **P < 0.01, ***P < 0.001, ****P < 0.0001

Left ventricular (LV) function were measured by echocardiography to assess the therapeutic effect of MSC-loaded GNR@SN/Gel in rat MI models at postoperative Day 28 (Fig. 5A).

Compared with the Sham group, all MI rats had decreased LV contractile function and increased LV internal diameter. The ejection fraction (EF) and fractional shortening (FS) of all MI rat groups were much lower than Sham group (all *P* < 0.05). While EF and FS in the MSCs/GNR@SN/Gel group were the highest among MI rats (Fig. 5B, C). The left ventricular internal diameter at end-systole (LVIDs) and left ventricular internal diameter at end-diastole (LVIDd) of all MI rat groups were much higher than Sham group (all *P* < 0.05). The MSCs/GNR@SN/Gel group demonstrated the smallest increase in the LVIDs compared with the other MI group (Fig. 5D, P < 0.05). LVIDd in MSC/GNR@SN/Gel group and MSC/SN/Gel group was much lower than other MI groups (Fig. 5E, P < 0.05), but no significant difference in LVIDd was observed between the MSC/GNR@SN/Gel group and MSC/SN/Gel group (Fig. 5E, P > 0.05).

### Evaluation of fibrosis by Masson trichrome staining and Sirius red staining

**Fig. 6:**
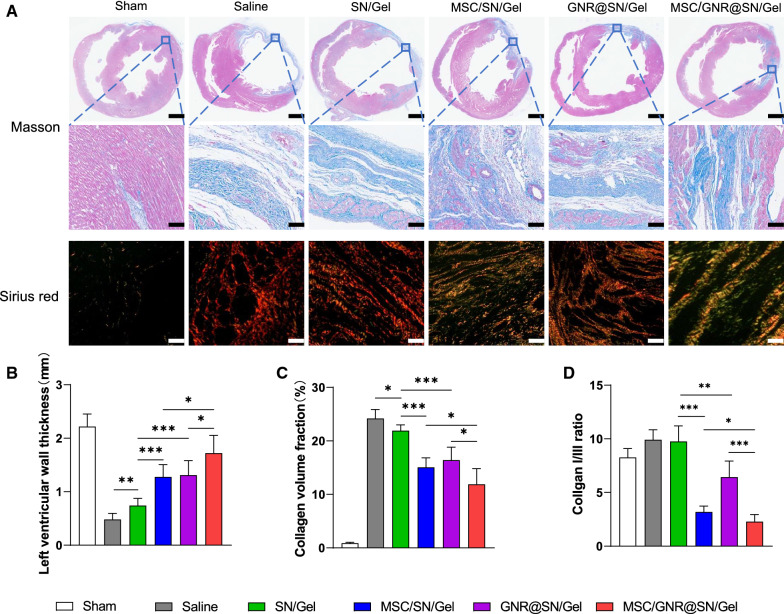
Evaluation of fibrosis by Masson trichrome staining and Sirius red staining at postoperative Day 28 and quantitative analysis. **A** Representative images of heart sections stained with Masson’s trichrome and Sirius red (Scale bars of heart cross sections of Masson’s trichrome staining: 1 mm, enlarged images of infarcted area: 100 µm; Sirius red: 100 µm). In Masson staining, collagen was stained blue; in Sirius red staining, the red and yellow tissues observed were considered type I collagen, while the green tissues observed were considered type III collagen. Quantitative analysis of the **B** ventricular wall thickness, **C** collagen content, and **D** collagen I/III ratio in different groups. *P < 0.05 and **P < 0.01, ***P < 0.001

MI often leads to excessive degradation of the extracellular matrix (ECM) and excessive collagen deposition, which is an important feature in cardiac remodeling [29]. Cardiac remodeling was observed using Masson trichrome staining. Infarcted areas exhibited different degrees of fibrotic tissue in different groups. The most severe degree of myocardial fibrosis was present in the Saline group, whereas the MSC/GNR@SN/Gel group had minimum fibrosis (Fig. 6A). The collagen volume fraction and LV wall thickness were calculated using Masson staining for quantitative comparison. Both the MSC/SN/Gel group and GNR@SN/Gel group showed a significant reduction in collagen volume fraction and significant increase in LV wall compared with the SN/Gel group. As expected, coadministration of the conductive injectable hydrogel and MSCs in the MSC/GNR@SN/Gel group contributed to the best inhibition profile against cardiac remodeling (Fig. 6A–C).

Scar tissue is composed of a variety of components, among which type I collagen is hard, while type III collagen has good elasticity [30]. Collagen I/III ratio can reflect the passive expansion ability of scar tissue. Sirius red staining was performed to observe collagen I and collagen III, the MSCs-encapsulated injectable hydrogel group (MSC/SN/Gel group, MSC/GNR@SN/Gel group) showed an extremely low collagen I/III ratio (Fig. 6A, D).

### Assessment of myocardial tissue electric-contraction coupling, revascularization and apoptosis

The structure of the electric-contraction coupling in the infarcted myocardium was analyzed by immunofluorescence staining. CX43 mediates electrical signaling between cardiomyocytes and functions by assembling into gap junctions between cells [31], α-actinin is one of the key myocardial skeleton proteins. Both proteins are downregulated in response to myocardial injury.

**Fig. 7:**
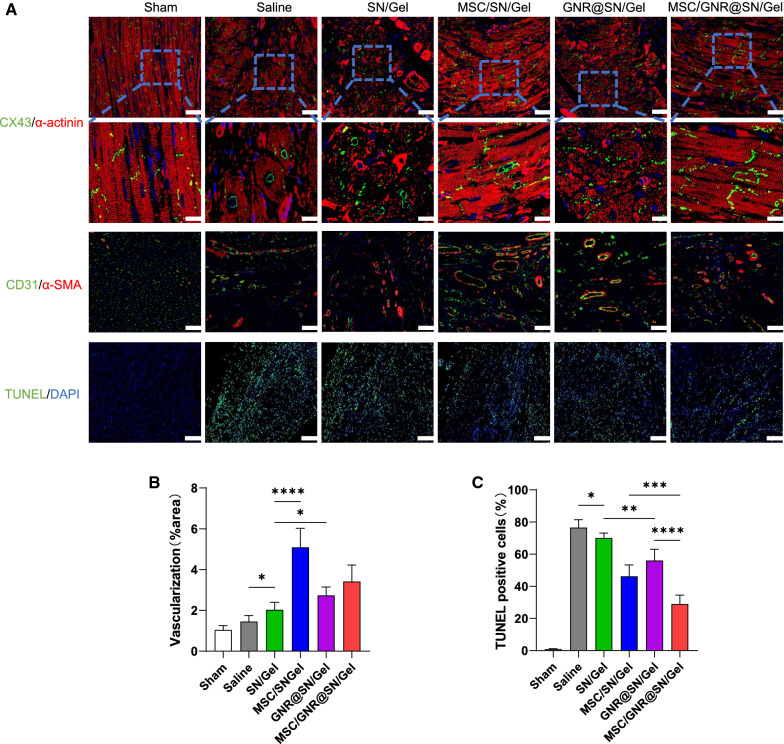
Assessment of myocardial tissue electric-contraction coupling, revascularization and apoptosis at postoperative Day 28. **A** Representative immunofluorescence images co-stained by CX43 (green) and α-actinin (red) (Scale bars: 50 μm, enlarged images: 20 µm); CD31 (green) and α-SMA (red), (Scale bars: 50 μm); TUNEL (green) and DAPI (blue) (Scale bars: 200 μm). Quantitative analysis of the **B** neovessels in the infarct regions and **C** cardiomyocyte apoptosis by TUNEL positive cells in normal myocardium adjacent to severely fibrotic tissue. *P < 0.05 and **P < 0.01, ***P < 0.001, ****P < 0.0001

In all MI groups, CX43 protein and α-actinin expression reduced most in the Saline group, while MSC/GNR@SN/Gel group exhibited the highest expression of CX43 (Fig. 7A). Furthermore, CX43 protein in the Saline group localized mainly at the periphery of the nucleus, whereas treatment of both MSC/SN/Gel and GNR@SN/Gel improved the functional localization of CX43 (Fig. 7A), and the MSC/GNR@SN/Gel group achieved the best treatment results, suggesting improved electromechanical coupling after treatment of MSC/GNR@SN/Gel.

Revascularization of myocardial structures restores blood supply to the ischemic myocardium. To assess the process of angiogenesis in ischemic myocardium, immunofluorescent staining for CD31 and alpha-smooth muscle actin (α-SMA), which jointly serve as markers of mature neovessels, were performed. Figure 7A, B reveal different degrees of angiogenesis were observed in all MI groups, except for the Sham group because angiogenesis does not occur in healthy myocardium. The Saline group showed a small amount of angiogenesis because of tissue hypoxia, and the SN/Gel group showed a slight increase compared with the Saline group. Angiogenesis in the GNR@SN/Gel group showed a significant increase compared with the SN/Gel group, which coincides with several animal experiments using conductive materials to treat MI [32, 33]. MSC/SN/Gel group showed the best angiogenic effect, followed by MSCs/GNR@SN/Gel group.

Myocardial apoptosis was assessed by the TUNEL assay and the site of cardiomyocyte apoptosis was observed in normal myocardium adjacent to severely fibrotic tissue. This experiment represents the apoptosis of myocardium caused by excessive compensatory contraction. The Sham group did not undergo MI operation, and almost no apoptosis was observed in the ventricular cardiomyocytes (Fig. 7A). Figure 7A, C revealed that the compensatory contract after MI operation caused more apoptosis of cardiomyocytes in the Saline group than other groups, and the MSC/GNR@SN/Gel treatment significantly alleviated apoptosis of cardiomyocytes.

## Discussion

The uniform dispersion of nanofillers in the polymer matrices is a general prerequisite for achieving desired mechanical and physical characteristics [34]. Because of their surface charge in solution, SNs can stabilize gold nanorods (GNRs) by electrostatic attraction. As a result, we used SNs as a sterically stabilized protective shield for GNRs and obtained uniformly dispersed and stabilized GNR dispersions in SNs (GNR@SN). SN as a crosslinker support the proliferation of MSCs [21], and PLGA-PEG-PLGA is a thermal-sensitive hydrogel can be crosslinked by SNs. Thus, we fabricated a cell-loadable thermal-sensitive conductive nanocomposite hydrogel fabricated by PLGA-PEG-PLGA, GNRs, and SNs. The advantages of this hydrogel in treating MI include: it is flowable at low temperature or at room temperature, giving it good injectable performance and easy doping of nanoparticles and cells. When injected into the body, it can be rapidly transformed into a gel phase, which exerts a mechanical support effect and also facilitates the local fixation of the therapeutic agent to the myocardium. Moreover, the temperature change is relatively mild and will not cause damage to cells.

Electrical integrity is essential for a well-functioning heart, and conductive biomaterials have been bioengineered to improve heart conduction and/or promote myocardial repair [35]. Recent key advancements have been centered on conductivity hypotheses of scaffolds to electronically bridge the gap between cardiomyocytes clusters thus yielding more robust phenotype [14]. High conductivity of SN/Gel and low conductivity of SN powder suggest that the conductivity imparted to the material by the SNs may arise mainly from the increased ionic concentration in the free interstitial water of the hydrogel due to exchangeable cations covered with SNs. The higher conductivity of GNR@SN/Gel compared with SN/Gel is due to GNRs stabilized by the crosslinker SNs form a unified whole via SNs with the tri-block polymer, allowing GNR@SN/Gel to increase the electronic conductivity of the polymer network. Although the conductivity of hydrogels can be improved by increasing the conductivity of polymer network scaffold and increasing the ion concentration of free interstitial water, one view is that ions in the solution will drift and diffuse to cancel this effect of conductivity on the cardiomyocytes [14], this is helpful to explain that although the conductivity of SN/Gel and GNR@SN/Gel are in the same order of magnitude, they have completely different effects on efficacy.

The efficacy of hydrogel as an implant is related to the correct implantation site, and the wrong implantation site can instead cause damage due to invasive treatment. Therefore, monitoring the location of hydrogel could noninvasively assess treatment and guide individualized precision therapy. For example, the information of the difference between the actual location of our hydrogel injections and the pre-determined location of the injections allows us to know whether the injected hydrogel was leaking into the heart chambers or outside the myocardium. SN as a cation exchanger has 47 meq./100 g ion exchange capacity [36]. We added ^68^Ga^3+^ into the hydrogel, cation adsorption capacity of SNs slowed down the release of ^68^Ga^3+^ in the hydrogel, which was helpful to identify the localization of GNR@SN/Gel in vivo with PET/CT imaging. ^68^Ga^3+^ has the advantages of easy availability through an ^68^Ge/^68^Ga generator and a short physical half-life of 67.71 min [37]. As a result, it is helps to understand the initial hydrogel placement without affecting ^18^F-FDG myocardial viability on the subsequent day.

Whether GNR improves the electrical conductivity of the material or the mechanical properties of the material, the efficacy of GNRs starts from the moment GNR@SN/Gel is injected into the heart, as demonstrated in our study that non-viable myocardium area in GNR containing groups (GNR@SN/Gel group and MSC/GNR@SN/Gel group) was significantly smaller than other groups on Day 1. MSC containing groups (MSC/SN/Gel group and MSC/GNR@SN/Gel group) revealed smaller expansion of non-viable myocardium than other MI group on Day 28, indicating the biological effects of MSC may occur later, the efficacy may be derived from the paracrine and immunomodulatory effects of MSCs [5–9]. Treatment with MSC/GNR@SN/Gel yielded the best results, with a protective effect on the myocardial viability on both Day 1 and Day 28, which was due to the combined therapeutic effect of GNR@SN/Gel and MSC.

As for the area expansion of non-viable myocardium, saline had no therapeutic effect, cardiomyocytes in ischemic myocardium were almost dead on the first day after MI operation in the Saline group. Subsequently, the infarct area of the Saline group continued to expand, and ventricular remodeling and dilation occurred at the same time, as a result, the percentage of non-viable myocardium (area of non-viable myocardium / total area of left ventricle) dilation was not obvious. However, statistical analysis revealed a significant difference between Day 1 and Day 28. In the treatment groups, hydrogel or MSCs provided different degrees of protection to the myocardium, thus the non-viable myocardium area on Day 1 in MSC/SN/Gel group, GNR@SN/Gel group, and MSC/GNR@SN/Gel group were significantly smaller than that in the Saline group. Subsequently, as the protective effect could not last long, apoptosis of myocardial cells in ischemic myocardium continued to occur, the non-viable myocardium area showed a trend of continuous expansion except MSC/GNR@SN/Gel group. However, the therapeutic components in this study were insufficient to reduce the size of the non-viable myocardium. There is still a long way to go in reducing non-viable myocardium.

The enhanced cardiac function observed in the MSC/GNR@SN/Gel group was likely because of the multiple roles of MSCs in treating MI, GNRs promoting electric signal propagation across the infarcted region, and improving the mechanical properties of the hydrogel. With the injectable hydrogel, the intramyocardial administration reinforced the mechanical stiffness of the myocardium, thereby attenuating myocardial remodeling and improving cardiac function [38, 39]. As a result, the SN/Gel group without MSCs and GNRs also preserved cardiac function compared with Saline group. Significant reduction in LVIDd was only observed after GNR@SN/Gel or SN/Gel combined with MSCs implantation. We speculated MSCs may play an important role in inhibiting the expansion of LVIDd.

Fibrosis is a key contributor to heart failure and its progression. However, fibrosis is more than just collagen deposition. It is generally defined as accelerated accumulation of ECM factors (predominantly collagen type I) and the replacement of apoptotic areas with collagen fibers, along with a progressive loss of elastic fibers [40]. After the loss of elastic fiber, the stiff fibrotic ventricular wall is unable to withstand pressure. Therefore, ventricular remodeling, such as enlargement of the infarcted area, ventricular dilatation, cardiomyocyte hypertrophy, and thinning of the ventricular wall occur [41]. As a result, the loss of elasticity of cardiac tissue is an important factor in promoting malignant remodeling of the myocardium. In patients with cardiomyopathy, increased collagen I, but not collagen III, is associated with systolic and diastolic dysfunction [30]. In this study, we observed that the MSCs-encapsulated injectable hydrogel group (MSC/SN/Gel group, MSC/GNR@SN/Gel group) showed an extremely low collagen I/III ratio and significant reduction in LVIDd (passive diastolic capacity of left ventricle). We speculated this may because MSCs have an ability to convert the composition of the scar [6–9], the higher composition of collagen type III with relatively good elasticity in cardiac scar tissue prevents the process of cardiac remodeling.

The vicious cycle between apoptosis and myocardial remodeling eventually leads to fatal heart failure, apoptosis in normal myocardium represents the decompensation of cardiomyocytes, which is accompanied by the depletion of large amounts of oxygen during the overload of cardiomyocytes [42]. TUNEL staining showed that the MSC/GNR@SN/Gel group was undergoing a relatively good remodeling process. Although MI induced the death of some cardiomyocytes, the MSC/GNR@SN/Gel group did not rapidly enter a series of subsequent vicious cycles that eventually led to fatal heart failure. Hypoxia is one of the most potent angiogenic stimuli [43], low oxygen consumption may be the reason why MSC/GNR@SN/Gel group did not show more angiogenesis than the MSC/SN/Gel group.

## Conclusions

We successfully developed a novel MSC-loaded conductive injectable hydrogel fabricated by GNRs, SNs, and PLGA-PEG-PLGA (GNR@SN/Gel), which is capable of MI therapy and monitoring of the implanted hydrogel. The combination of MSCs and this conductive hydrogel improved electromechanical coupling, exhibited a protective effect on the myocardial viability in early and late stages, and improved cardiac function. We believe MSCs combined with GNR@SN/Gel therapy is a potential strategy for MI treatment.

## Methods

### Synthesis and characterization of the hydrogel

PLGA_2000_-PEG_3400_-PLGA_2000_ was provided by Xi'an Rui Xi Biotechnology Co Ltd (Xi'an, China). The average molecular weight was determined using 1H-NMR (JEOL, ECA-500; solvent, CDCl3). SNs (Laponite XLG) was provided by BYK (Wesel, Germany). PLGA_2000_-PEG_3400_-PLGA_2000_ was dissolved in acetone and pure water was added. The acetone was completely evaporated to prepare an aqueous solution of PLGA_2000_-PEG_3400_-PLGA_2000_. The solution was stored in a freezer at 4 °C for 1 h for later use. Thereafter, SNs (2% w/v) was added to the GNR dispersion and the samples were vortexed for 30 s and sonicated on ice three times for 20 s each. After thorough mixing, PLGA_2000_-PEG_3400_-PLGA_2000_ solution was added, stirred vigorously for 120 s and then incubated at 4 °C for 10 min to prepare the dual nanocomposite conductive hydrogel precursors [1% (w/v) SNs, 3% (w/v) PLGA_2000_-PEG_3400_-PLGA_2000_, 0.3% (w/v) GNRs]. The temperature-induced sol–gel phase transition behaviors of GNR@SN/Gel or SN/Gel were measured using a rheometer (DHR-2, Waters, America) with a cone plate (1° steel cone, 60 mm diameter). The temperature was increased from 15 to 40 °C, and time sweep program was set at an oscillatory frequency of 10 rad/s and heating rate of 0.5 °C/min. Swelling experiments were performed by immersing the as-prepared hydrogels in a large excess of deionized water at 25 °C for 1 day to reach swelling equilibrium. The swollen samples were then freeze-dried. The swelling ratio (Q) was evaluated using Q = Ws/Wd, where Ws and Wd are the weights of the swollen sample and the corresponding dried xerogel sample, respectively. For characterization of gel degradation, the hydrogels were formed and incubated in culture media at 37 °C for 1, 3, 7, 14, and 21 days. Then, the hydrogels were removed from the incubation medium, frozen, and lyophilized. The dry mass of the hydrogels was measured following lyophilization. To correlate the effect of SNs to the adsorption capability, GNR@SN/Gel and SN/Gel were studied. Previous to adsorption, 0.3 mL of the hydrogel precursor was cross-linked by exposure to a water bath at 37 °C. Samples (n = 3) were soaked in a FITC-labeled BSA solution for 1, 2, 4, 8, and 24 h. Supernatant was collected to analyse adsorption by microplate reader (Bio-Tek Instruments Inc., Winooski, VT, USA).

### In vitro cell experiment

#### Live/dead assay

Live/dead assay was performed using a Live/dead cell assay Kit (Solarbio, China). Cell-loaded hydrogel was incubated in buffer from the kit with 2 μM calcein AM and 8 μM PI for 1 h per manufacturer’s protocol. After 1 h incubation at 37 °C, the hydrogel was washed three times with buffer, and fluorescent images were obtained using inverted fluorescent microscope.

#### Treatment of rats’ MI with the hydrogel

Animal experiments were approved by Ethics Committee of Union Hospital, Tongji Medical College, Huazhong University of Science and Technology. All animal experiments were conformed to the guidelines and standards of the Experimental Animal Center of Tongji Medical College, Huazhong University of Science and Technology.

Sprague Dawley (SD) rats, male, weighing ~ 200 g, were purchased from the Experimental Animal Center in Tongji Medical College. After the rats were anesthetized and artificially ventilated with a small animal ventilator, the left anterior descending coronary artery was ligated with 6.0 prolene nonabsorbable suture. The ligation was deemed successful when the anterior wall of the LV turned pale. The injection procedure of the MSCs-loaded hydrogel was performed as follows: after successful ligation of rats, the MSCs cultured in the incubator were immediately digested with trypsin for 3 min, after trypsin digestion of the adherent cells, the cells were collected by centrifugation at 1000 rpm. Carefully remove as much supernatant as possible with a pipette, resuspension with saline, and perform a cell count, a quantitative number of cells were removed and centrifuged, then the supernatant was removed as much as possible, and the prefabricated hydrogel precursor was mixed with MSC and blown evenly. On the basis of ligation of rats, hydrogel (90 μL) was transplanted to the infarcted myocardium 30 min after coronary ligation. After injections, the chests were closed and animals recovered on a heating pad.

### Image-guided delivery and assessment of the therapeutic effect

#### Assessment the delivery of hydrogel

After MI operation, ^68^GaCl_3_ was encapsulated in hydrogel (50 µCi/mL) and in situ injected into rat heart. After chest closure and postoperative care, PET/CT scan was performed to observe ^68^Ga-loaded injectable hydrogel in the heart of rats on a dedicated small animal PET/SPECT/CT system (Inliview-3000B, Beijing, Novel Medical Equipment Ltd.).

#### Myocardial viability assessment by ^18^F-FDG myocardial PET imaging

Rats underwent prior fasting over 14 h. One hour before ^18^F-FDG injection, rats received 5%w/t dextrose in water ad libitum. One hour after tail vein injection of ~ 0.2 mCi ^18^F-FDG, 10 min static PET images were acquired. Anesthesia of the rats was started 10 min before PET acquisition, and all rats were kept anesthetized with 1.5% isoflurane throughout the imaging procedure.

#### PET image analysis

PET images were processed by PMOD 3.9 software. A visualized 17-segment polar map of rat myocardial viability was obtained to outline the rat ventricles. The cut-off is 40% of the highest value of FDG uptake by the myocardium, and the region with uptake values below 40% is considered non-viable myocardium. For ^68^Ga PET images, 17 segments were directly mapped to the image of ^68^Ga-loaded hydrogel to obtain the location of injected hydrogel in rat ventricular segment.

#### Echocardiographic assessment

The GE vivid 7 system equipped with a 10 MHz transducer was used to assess left ventricular function in rats 28 days after surgery. Isoflurane anesthesia was used and echocardiograms were obtained. Transthoracic two-dimensional guided m-tracking was used to obtain parasternal long-axis views at the level of the papillary muscles. Left ventricular ejection fraction (EF), short systolic fraction (FS), left ventricular internal diastolic diameter (LVIDd), and left ventricular internal systolic dimensions (LVIDs) were calculated as the mean of three consecutive cardiac cycles.

### Histological evaluation

#### Tissue sample collection

The rats were sacrificed and their hearts were collected. Samples of ventricular myocardium were excised, fixed in 4% paraformaldehyde, embedded in paraffin, cut into 5 μm-thick sections, and mounted on slides.

#### Masson trichrome-staining

Masson-trichrome staining was performed with a Masson Trichrome Stain Kit (Sigma-Aldrich) to evaluate the collagen volume fraction and the LV wall thickness, the collagen volume fraction was calculated as the ratio of the total area of fibrosis to the myocardium in the entire specimens. Collagen volume fraction were quantified via ImageJ software*.*

#### Sirius red staining

Sirius red staining was performed using Sirius red and picric acid obtained from Sigma. Sections were visualized under polarized light. Collagen I/III ratio were quantified using ImageJ, the red and yellow tissues were identified as collagen type I and the green as collagen type III, 5 fields (200×) were analyzed per specimens*.*

#### Immunofluorescence staining

Immunofluorescence staining for CX43, α-actin, α-SMA and CD31 was performed on paraffin-embedded sections. Slides were permeabilized with PBS (pH = 7.4) for 40 min, gently shaken three times on a decolorization shaker, slightly dried, and blocked with 3% BSA for 30 min at room temperature. The primary antibody was added and samples incubated at 4 °C overnight. After that, secondary antibody was added for 1 h incubation at room temperature. After PBS rinsing, nucleus was stained using DAPI. TUNEL staining was performed according to the manufacturer’s directions (Roche, Germany), and analyzed using the ImageJ software*.*

### Statistical analysis

Data were processed and analyzed using GraphPad Prism 9.1 (GraphPad). The data are presented as mean ± standard deviation. One-way analysis of variance analysis (ANOVA) or paired t test was used to compare the differences between groups. *P* < 0.05 were considered significant.

## Supplementary Information

**Additional file 1.** Additional Methods includes synthesis of the gold nanorods, tube inversion method, MSCs extraction and identification and ^68^Ga^3+^ release rate of different samples. **Figure S1.** The molecular composition of PEG, dl-LA, and GA in the obtained copolymers was estimated by 1H-NMR measurements. **Figure S2.** The SN/Gel (3% w/v PLGA_2000_-PEG_3400_-PLGA_2000_, 1% w/v SN) hydrogel precursor can be transformed into a gel at 37 °C while remaining a flowable sol phase at 28 °C, while GNR@SN/Gel transforms gel phase at 28 °C. **Figure S3.** Molecular markers of MSCs were detected by flow cytometry. **Figure S4.** ^68^Ga^3+^ release rate of different samples.

## Data Availability

Not applicable.

## Abbreviations

MI: Myocardial infarction
MSCs: Mesenchymal stem cells
SNs: Silicate nanoplates
GNRs: Gold nanorods
PLGA-PEG-PLGA: Poly(lactide-*co*-glycolide)-*b*-poly (ethylene glycol)-*b*-poly(lactide-*co*-glycolide)
BSA: Bovine albumin
PBS: Phosphate buffered saline
CX43: Connexon 43
α-SMA: Alpha-smooth muscle actin
