# Supplementary material for: Conductive nanocomposite hydrogel and mesenchymal stem cells for the treatment of myocardial infarction and non-invasive monitoring via PET/CT
Source: J Nanobiotechnology. 2022 May 6;20:211. doi: 10.1186/s12951-022-01432-7 (PMC9077894; doi:10.1186/s12951-022-01432-7)
Supplement: Supplementary file 1 — Additional file 1. Additional Methods includes synthesis of the gold nanorods, tube inversion method, MSCs extraction and identification and 68Ga3+ release rate of different samples. Figure S1. The molecular composition of PEG, dl-LA, and GA in the obtained copolymers was estimated by 1H-NMR measurements. Figure S2. The SN/Gel (3% w/v PLGA2000-PEG3400-PLGA2000, 1% w/v SN) hydrogel precursor can be transformed into a gel at 37 °C while remaining a flowable sol phase at 28 °C, while GNR@SN/Gel transforms gel phase at 28 °C. Figure S3. Molecular markers of MSCs were detected by flow cytometry. Figure S4. 68Ga3+ release rate of different samples. [file 12951_2022_1432_MOESM1_ESM.docx]

Additional file 1

**Title page**

**Conductive nanocomposite** **hydrogel and mesenchymal stem cells for the treatment of myocardial infarction and non-invasive** **monitoring via PET/CT**

*Ke Zhu^1,2#^, Dawei Jiang^1,2#^, Kun Wang ^1,2^, Danzha Zheng^1,2^, Ziyang Zhu^1,2^, Fuqiang Shao^1,2^, Ruijie Qian^1,2^, Xiaoli Lan^1,2^, and Chunxia Qin*^1,2^*

^1^ Department of Nuclear Medicine, Union Hospital, Tongji Medical College, Huazhong University of Science and Technology, Wuhan 430022, China

^2^ Hubei Key Laboratory of Molecular Imaging, Wuhan 430022, China

**# These authors contributed equally to this work.**

***Correspondence**: qin_chunxia@hust.edu.cn

**Additional Methods**

**Synthesis of the gold nanorods**

Gold nanorods (GNRs) were synthesized by a seed-mediated approach. Briefly, an aqueous solution of 25 mM freshly prepared HAuCl_4_·3H_2_O and 0.1 M CTAB (7.5 ml) was gently stirred. Subsequently, 600 μL of ice-cold NaBH_4_ solution (10 mM) was added and mixed for 2 min. The obtained brown-yellow seed solution was aged at 25 ℃ for more than 2 h before use. Next, the GNRs growth solution consisting of 0.1 M CTAB (100 mL), 25 mM HAuCl_4_ (2.04 mL), 0.5 M H_2_SO_4_ (2 mL), 0.01 M AgNO_3_ (0.9 mL) and of 0.1 M l-ascorbic acid (0.8 mL) was prepared. The obtained seed solution (240 μL) was added to the growth solution and gently stirred for 45 min. The solution was then kept at room temperature overnight without stirring. GNRs were purified by centrifuging at 11,000 rpm for 15 min, washed at least three times with distilled water, and finally dispersed in distilled water for further application.

**Tube inversion method**

Critical transition temperature of the hydrogel was determined by tube inversion method. Briefly, each formulation was sealed in a small tube and heated slowly from 10°C to 40°C at a rate of 1°C/min. The sample was equilibrated for 2 min at each temperature point, and the flowability of each sample was observed by tilting the tubes. The temperature at which the liquid was observed to be immobile was recorded as the critical transition temperature.

**MSCs extraction and identification**

Male SD rats weighing approximately 50g were sacrificed by spinal cord dissection. Bilateral femurs were harvested under aseptic conditions, and all muscle tissues were removed. The bone marrow cavity was irrigated with Dulbecco’s modified eagle medium (DMEM, HyClone, USA) to obtain a mixture of bone marrow and DMEM. After centrifugation, the precipitate was resuspended in DMEM + bovine serum albumin (BSA, HyClone) + penicillin-streptomycin (Solaibao, Beijing, China). Culture was conducted for 3 days under the following conditions: 95% humidity, 37 ℃ temperature and 5% carbon dioxide concentration. The non-adherent cells were then removed, and the culture medium was refreshed. After the adherent cells reached 90–95% confluence, the cells were passaged, and the third passage (P3) of stem cells was used for identification and subsequent experiments. The surface markers CD44, CD90, CD34, and CD31 (antibodies from Abcam) were detected by flow cytometry.

**^68^Ga^3+^ release rate of different samples**

The aqueous polymer solutions containing ^68^Ga^3+^ (50μCi/mL) were readily prepared by blending the aqueous polymer solution with GaCl_3_ at 4 °C to form a homogenous solution. The ^68^Ga^3+^-loaded aqueous polymer solution maintained a free-flowing sol state at room temperature while a gel state at body temperature. The samples (1 mL) were injected into 4 mL vials, equilibrated at 4 °C for 5 min, and then incubated in a water bath at 37 °C for 5 min to form gels. 1mL PBS was added to the vials, and then incubated in a water bath at 37 °C for 60 min. The ^68^Ga^3+^ contents in the PBS samples were detected by radioactivity measurement. The PLGA_1800_-PEG_1500_-PLGA_1800_ hydrogel used as the control group was purchased from Shanghai Yuanye Bio-Technology Co Ltd.


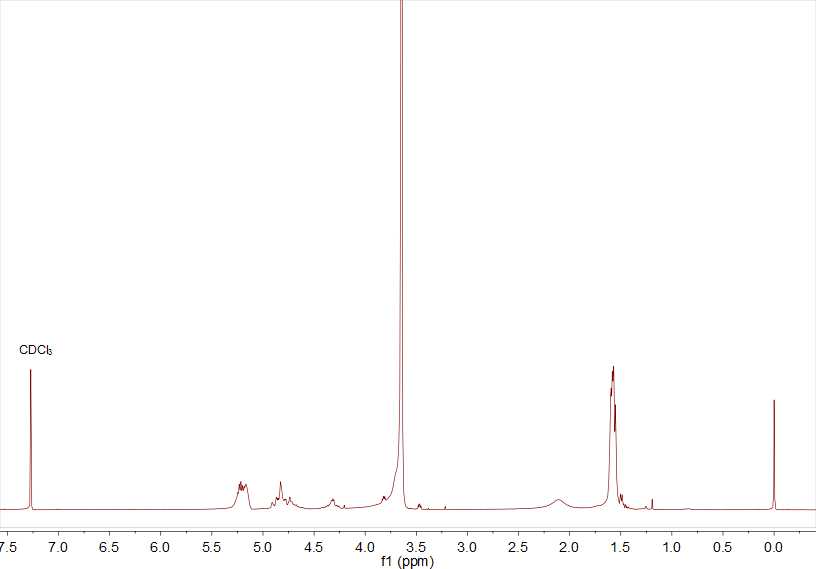


**Figure S1.** The molecular composition of PEG, dl-LA, and GA in the obtained copolymers was estimated by ^1^H-NMR measurements.


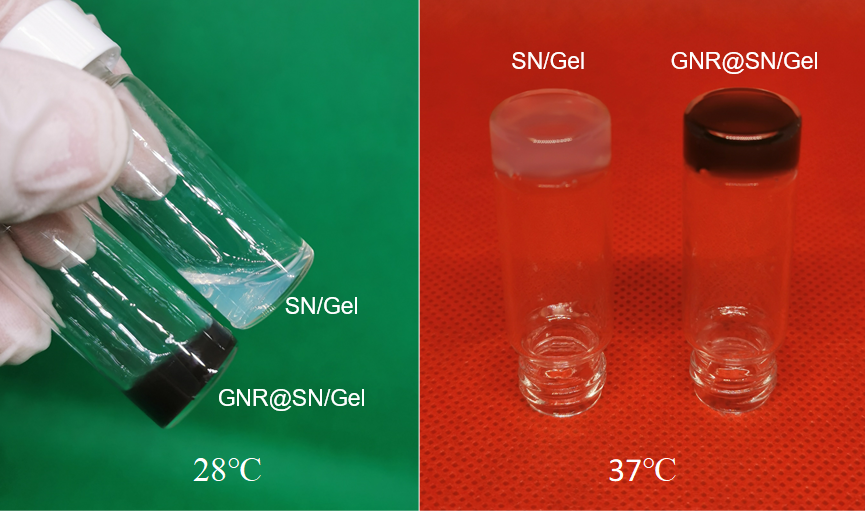


**Figure S2.** The SN/Gel (3% w/v PLGA_2000_-PEG_3400_-PLGA_2000_, 1% w/v SN) hydrogel precursor can be transformed into a gel at 37 ℃ while kept in a flowable sol phase at 28 ℃, while GNR@SN/Gel transforms gel phase at 28 ℃.

**Figure S3.** Molecular markers of MSCs were detected by flow cytometry.


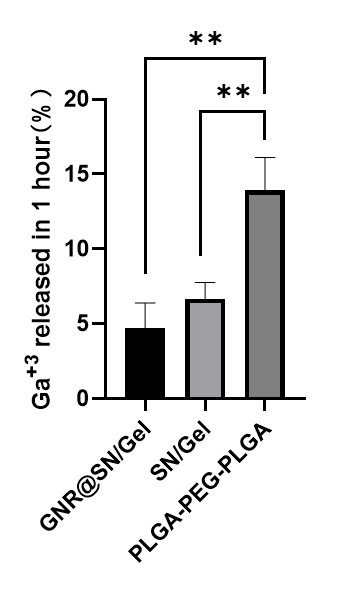


**Figure S4.** ^68^Ga^3+^release rate of different samples.
